# Pathology of Podocytopathies Causing Nephrotic Syndrome in Children

**DOI:** 10.3389/fped.2016.00032

**Published:** 2016-03-31

**Authors:** Sarangarajan Ranganathan

**Affiliations:** ^1^Department of Pathology, Children’s Hospital of Pittsburgh of UPMC, Pittsburgh, PA, USA

**Keywords:** congenital nephrotic syndrome, Finnish type, diffuse mesangial sclerosis, minimal change disease, focal segmental glomerulosclerosis, C1q nephropathy

## Abstract

Nephrotic syndrome (NS) in children includes a diverse group of diseases that range from genetic diseases without any immunological defects to causes that are primarily due to immunological effects. Recent advances in molecular and genomic studies have resulted in a plethora of genetic defects that have been localized to the podocyte, the basic structure that is instrumental in normal filtration process. Although the disease can manifest from birth and into adulthood, the primary focus of this review would be to describe the novel genes and pathology of primary podocyte defects that cause NS in children. This review will restrict itself to the pathology of congenital NS, minimal change disease (MCD), and its variants and focal segmental glomerulosclerosis (FSGS). The two major types of congenital NS are Finnish type characterized by dilated sausage shaped tubules morphologically and diffuse mesangial sclerosis characterized by glomerulosclerosis. MCD has usually normal appearing biopsy features on light microscopy and needs electron microscopy for diagnosis, whereas FSGS in contrast has classic segmental sclerosing lesions identified in different portions of the glomeruli and tubular atrophy. This review summarizes the pathological characteristics of these conditions and also delves into the various genetic defects that have been described as the cause of these primary podocytopathies. Other secondary causes of NS in children, such as membranoproliferative and membranous glomerulonephritis, will not be covered in this review.

## Introduction

The kidney is the main organ of filtration in the body, and the daily protein loss is only a small portion of the total protein ingested. Nephrotic syndrome (NS) is characterized by heavy proteinuria that exceeds 1.66 g/1.73 m^2^/day in children, edema, hypoalbuminemia, and hyperlipidemia (1). Although the causes of NS are many and diverse, it is a frequent cause of renal disease in children with an annual incidence of about 2 to 7 children per 100,000 (1). The International Study of Kidney Disease in Children lists minimal change disease (MCD) as the commonest cause of primary NS in children affecting 77% of the children followed by focal segmental glomerulosclerosis (FSGS) at about 8% followed by membranoproliferative glomerulonephritis (MPGN) and membranous glomerulonephritis (2). The causes of NS also vary to some extent with the age of presentation with the congenital forms more directly associated with specific genetic defects and those appearing in later childhood related more often to secondary causes.

## Pathophysiology of NS

The primary defect in NS is loss of proteins in the kidney. Although lack of tubular reabsorption could lead to proteinuria, NS range proteinuria usually implies permeability defects in the glomerular membrane that results in this excessive protein loss. This leads to albuminuria and hence the associated hypoalbuminemia and edema, the two main manifestations of NS. The hyperlipidemia is usually due to the increased lipoprotein synthesis induced by the hypoalbuminemia, and this may lead to increased platelet aggregation and thrombosis, one of the complications of NS. The loss of other proteins, minerals, and vitamins with the proteinuria may also predispose to malnutrition and infections. The most dramatic advances for understanding the pathophysiology of NS has occurred in the area of podocyte biology and the structure of the slit diaphragm (3–5). The glomerular filtration barrier consists of the fenestrated capillary endothelium, the extracellular basement membrane, and the intercalated podocyte foot processes. NS is associated with the biopsy finding of effacement of podocyte foot processes. Effacement is characterized by flattening of the podocyte, retraction of foot processes, and sometimes microvillous transformation. It has also been understood that MCNS and FSGS can be classified as podocytopathies, in which disruption of slit diaphragm and normal podocyte function can lead to proteinuria and glomerular disease. Details of the structure and mechanisms of podocyte injury and defects are beyond the scope of this article, and readers are directed to several reviews on podocytes (3–5).

## Causes of Nephrotic Syndrome in Children

The causes of NS show some variations depending on the age of the child with early onset NS usually representing primary genetic or idiopathic causes, such as congenital NS, due to any of the known genetic defects or MCD and less often FSGS (Table 1) (2, 6, 7). As the age increases, the ratio of MCD and FSGS may vary with secondary causes becoming more common in the second decade. Although NS may be the only presentation in children, often the condition is precipitated or can be seen as part of a nephritic syndrome. There is still some controversy on whether MCD and FSGS are related entities with MCD representing the early form of a disease and FSGS the late stage. This is especially so since there is often the finding of an early biopsy with MCD changes with refractory disease and a later biopsy showing features of FSGS. Also, while several genes have now been identified in MCD and FSGS, no distinct genetic defects have been found for these two diseases (7).

**Table 1:** **Causes of nephrotic syndrome in children**.

**Genetic**
Congenital nephrotic syndrome of Finnish type
Diffuse mesangial sclerosis (DMS)
Isolated DMS
Part of Denys–Drash syndrome
Epidermolysis bullosa associated
Steroid-resistant nephrotic syndrome
Familial focal segmental glomerulosclerosis (FSGS)
**Infectious causes**
Congenital infections including syphilis, toxoplasmosis, and HIV
Cytomegalovirus
HIV-associated nephropathy
**Idiopathic**
Minimal change nephropathy
Focal segmental glomerulosclerosis
Diffuse mesangial hypercellularity
Membranous glomerulonephritis
Membranoproliferative GN (MPGN) (NS may predominate or with nephritic syndrome)
**Others**
Lupus nephropathy
IgA nephropathy
Drugs
Malignancies
Hemolytic uremic syndrome (HUS)

## Genetics of NS

**Figure 1:**
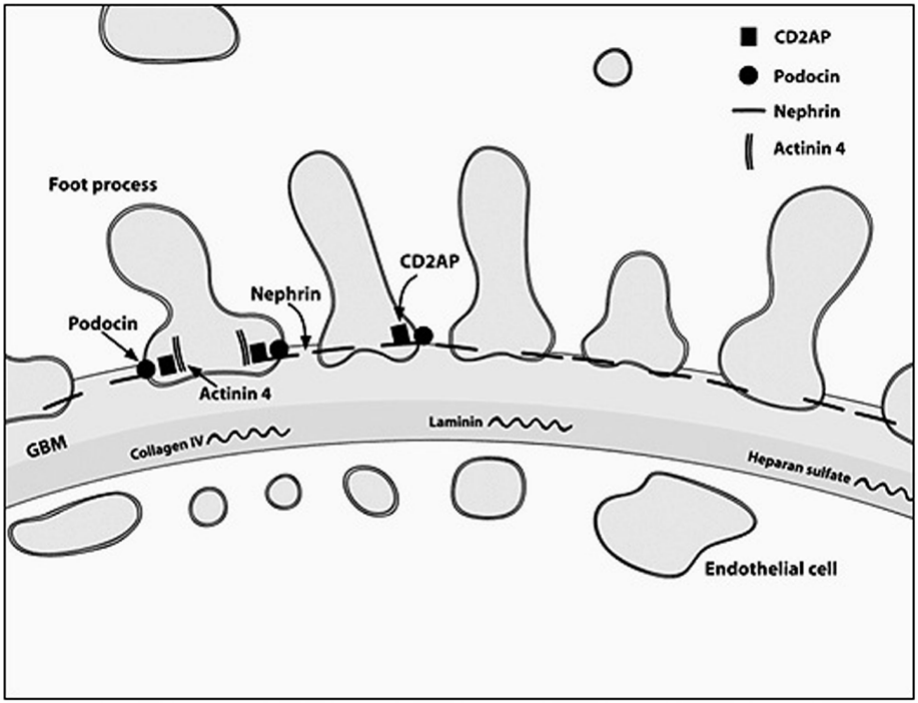
**A diagrammatic representation of the basement membrane foot processes with the location of the most common genes implicated in nephrotic syndrome**.

The podocyte, as mentioned before, is the critical structure and has a critical role in pathogenesis of NS (Table 2; Figure 1). Podocytes form foot processes and slit diaphragms as part of normal development. This leads to the expression of specific proteins, such as ZO-1, in the slit diaphragm and nephrin, podocin, and CD2AP (adhesion molecule CD2-associated protein) are expressed (5). Nephrin gene mutations cause Finnish-type nephropathy (7, 8). Other developmental genes involved in podocyte structure and function include Pax-2 and WT1. The WT1 gene mutation results in the Denys–Drash syndrome and Frasier syndrome. Podocin gene defects cause familial autosomal-recessive steroid-resistant NS in early childhood. The individual genetic defects are described further in the respective disease sections. It is critical to understand the structure of the podocyte and the basement membrane with all its collagen to understand the genetic defects and their potential effect. Discovery of novel mutations has led to further understanding of the biology and prognosis of these diseases.

**Table 2:** **Genetic causes of nephrotic syndrome**.

Genes	Location (if known)	Protein	Significant clinical association
NPHS1	19q13.12	Nephrin	CNS Finnish type
NPHS2	1q25.2	Podocin	Steroid-resistant NS; rapidly progressive renal disease; FSGS
WT1 (NPHS4)	11p13	Wilms’ tumor 1	Denys–Drash syndrome; nephrotic syndrome–FSGS; Frasier syndrome
SMARCAL1	2q35	SW1/SNF related	Schimke immunoosseous dysplasia
PLCE1 (NPHS3)	10q23.33	Phospholipase C_E_1	DMS, FSGS
PTPRO	12p12.3	Protein tyrosine phosphatase, receptor-type O	Steroid-resistant NS
LAMB2	3p21.31	Laminin, beta-2	CNS with ocular abnormalities; Pierson syndrome
INF2 (FSGS 5)	14q32.33	Inverted formin 2	FSGS
COQ6	14q24.3	Coenzyme Q10 def, primary 6	Progressive NS in infancy with sensorineural deafness; FSGS, DMS
MYO1E (FSGS6)	15q21	Myosin 1E	FSGS (AR)
TRPC6 (FSGS2)	11q22.1	Transient receptor potential cation channel, subfamily C, member 6	FSGS (AD)
COQ2	4q21.23	Coenzyme Q10 deficiency-1	Steroid-resistant NS
LMX1B	9q33.3	LIM homeobox transcription factor 1, beta	Nail–patella syndrome
ADCK4 (NPHS9)	19q13.2	AARF domain-containing kinase 4	NS (AR)
PDSS2	6q21	Prenyl diphosphate synthese, subunit 2	NS
ACTN4 (FSGS1)	19q13.2	Alpha-actinin-4	FSGS
CD2AP (FSGS3)	6p12.3	CD2-associated protein	FSGS
MYH9	22q13.1	Non-muscle myosin IIA heavy chain	FSGS, collapsing glomerulopathy

## Role of Renal Biopsy in Diagnosis of NS

In many instances, there may be no role for pathologist for diagnosis due to the ability to do genetic testing for individual mutations and due to the response to steroid therapy, but increasing incidence of resistance to steroid therapy has increased the role of the pathologist in determining if FSGS is the cause of steroid resistance. Other causes for biopsy are presence of coexisting nephritic syndrome and for determination of other causes of NS. All renal biopsies need triaging for adequacy to perform immunofluorescence and electron microscopy (EM). In view of the broad etiology and associated conditions, the pathologist very often has a critical role in guiding therapy in these patients. Routine histology warrants a PAS and silver stain for assessment of basement membranes and a Masson trichrome stain for assessment of fibrosis and glomerulosclerosis as well as vascular changes. The standard panel for IF includes IgG, IgA, IgM, C3, C4, C1q, and albumin with kappa and lambda where needed. EM is necessary for diagnosis in many instances.

## Pathology of Common Causes of NS in Childhood

### Congenital Nephrotic Syndrome of Finnish Type

**Figure 2:**
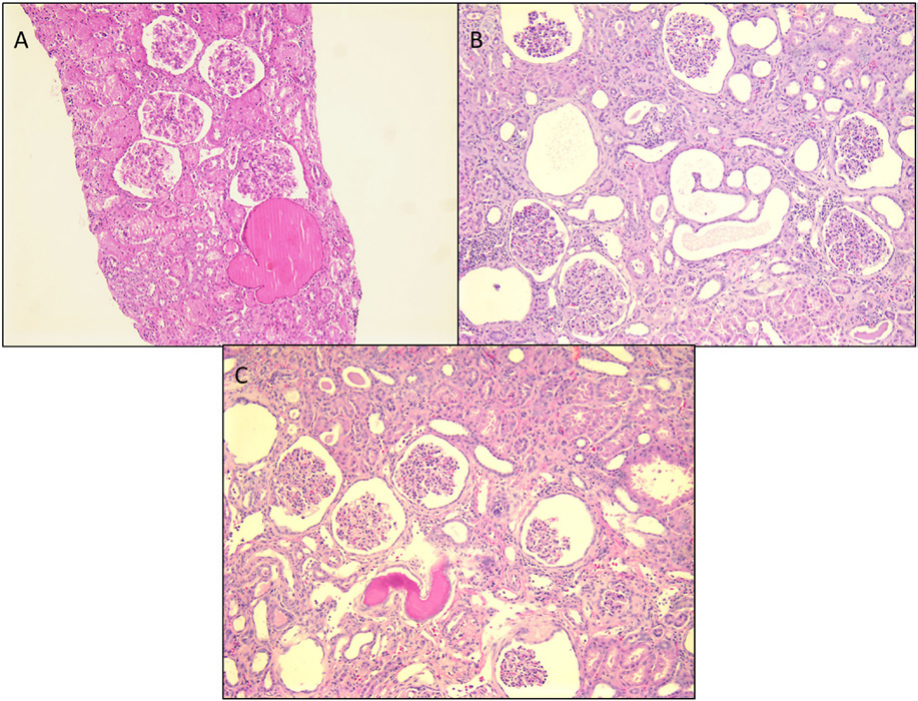
**Histological panel for congenital nephrotic syndrome of Finnish type**. **(A)** A photomicrograph showing a cluster of normal appearing glomeruli with dilated proximal tubules with proteinaceous contents (H&E 100×). **(B)** Another area from this resected renal specimen showing the varying shapes of the dilated proximal tubules, a characteristic feature of congenital NS (H&E 100×). **(C)** Another image from the opposite kidney showing the same morphological features with no segmental sclerosis evident at this time (H&E 100×).

It is an autosomal-recessive disorder with an incidence of 1:8200 births in Finland (9). It is also seen in North American and European populations and is a cause of congenital NS with prenatal presentation in many cases. Diagnosis has been made *in utero* by amniocentesis that shows elevated alpha-fetoprotein as early as 16–18 weeks gestation (10). It results in small for gestational age neonate with prematurity, deformities due to contractures and an abnormally enlarged placenta. It results in heavy proteinuria in the neonatal period with selective proteinuria in the early stages and more non-selective proteinuria in the later stages as the disease progresses. Most infants do not survive beyond 1 year of life, usually due to infections and sepsis related to loss of immunoglobulins. The gene for Finnish nephropathy (FN) is *NPHS1* mapped to the long arm of chromosome 19 (19q13.1) that codes for nephrin (7, 11, 12). Several nephrin mutations have now been identified. The typical mutations are a 2-bp deletion in exon 2 (Fin-major) or a nonsense mutation in exon 26 (Fin-minor). Renal biopsy shows normal glomeruli or some with mesangial hypercellularity, hyperlobulated capillary tufts, and some scarring. Microcystic dilatation of proximal and distal tubules is also seen, and there may be associated interstitial fibrosis and inflammation (Figure 2). Immunofluorescence is negative, whereas EM shows diffuse foot process effacement with or without villous transformation. Immunohistochemical stain for nephrin, now commercially available will show negative staining of podocyte. This stain helps in differentiating FN from other causes of congenital NS. Development of anti-nephrin antibodies and recurrence in allograft kidney has been reported (13).

### Diffuse Mesangial Sclerosis

**Figure 3:**
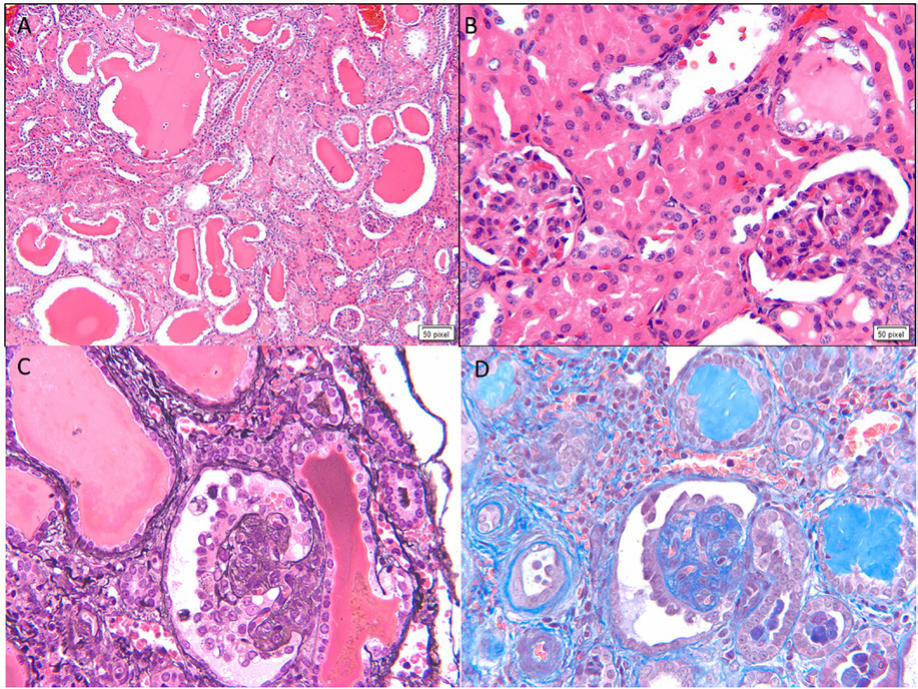
**Histological images for diffuse mesangial sclerosis**. **(A)** An image from another nephrectomy specimen showing the numerous dilated tubules with small occasional glomeruli (H&E 40×). **(B)** Higher magnification of the glomeruli showing a solidified appearance on light microscopy (H&E 200×). **(C)** A Jones silver stain highlighting the solidified loops with accentuation of the epithelial cells on the surface. No capillary loops are identified (Jones methanamine silver 200×). **(D)** A trichrome stain showing the mesangial sclerosis characteristic of this disease. Note again the prominent epithelial cells. Progressive disease leads to glomerular obsolescence and interstitial fibrosis seen in this image (Trichrome 200×).

This is the second most common cause of congenital NS diagnosed on renal biopsy. Although the presentation is similar to that of FN, diffuse mesangial sclerosis (DMS) can present later up to 4 years of age. It presents with unremitting NS within the first 9 months of life usually. Infants develop hypertension with rapid onset of renal failure. The combination of DMS, Wilms tumor, and male pseudohermaphroditism constitutes Denys–Drash syndrome (14). Other associations include cataract, strabismus, nystagmus, myopia and aniridia, mental retardation, microcephaly, deafness, musculoskeletal abnormalities, and cleft palate. DMS is also part of the Galloway–Mowat syndrome, Pierson syndrome (LAMB2 mutations), and Frasier syndrome (7). It is caused by WT1 gene mutations in exon 8 or 9 (14). Frasier syndrome is usually caused by a splice variant mutation in exon 9 (15). The earliest light microscopic feature is increased mesangial matrix that is global and diffuse. There is a gradient of changes seen from the outer to the inner cortex with the most severe sclerosis being seen in the outer cortex, DMS in the mid zone and milder sclerosis in the inner zone (Figure 3). In some cases, prominent focal or global glomerulosclerosis may be seen. Podocytes may be prominent over the tufts. Severe tubulointerstitial damage with cysts and tubular ectasia may be seen. Immunofluorescence shows no immune deposits or non-specific mesangial IgM, C3, and C1q, whereas EM shows variable effacement of foot processes. Glomerular basement membrane lamellations and splitting similar to Alport’s syndrome may be seen. In late stages, there is widening of the BM with thickened capillary loops and finally a sclerotic glomerulus.

### Minimal Change Disease

Minimal change disease is more common in boys than girls with a ratio of 2:1. Almost 80% of cases occur in children <6 years of age (median age 2.5 years) (1, 16). It can be idiopathic, secondary, or familial. The most common form is sporadic MCD that is usually steroid sensitive. The genes mutated in MCD include NPHS1 and NPHS 2 (7). Mutations in the dysferlin gene have also been described. More recently, mutations in epithelial membrane protein 2 (EMP2) gene have been shown to cause childhood-onset NS. Crumbs homolog 2 (CRB2) defects have been associated with steroid-resistant NS. NPHS2 mutations have been associated with steroid resistance (17, 18).

**Figure 4:**
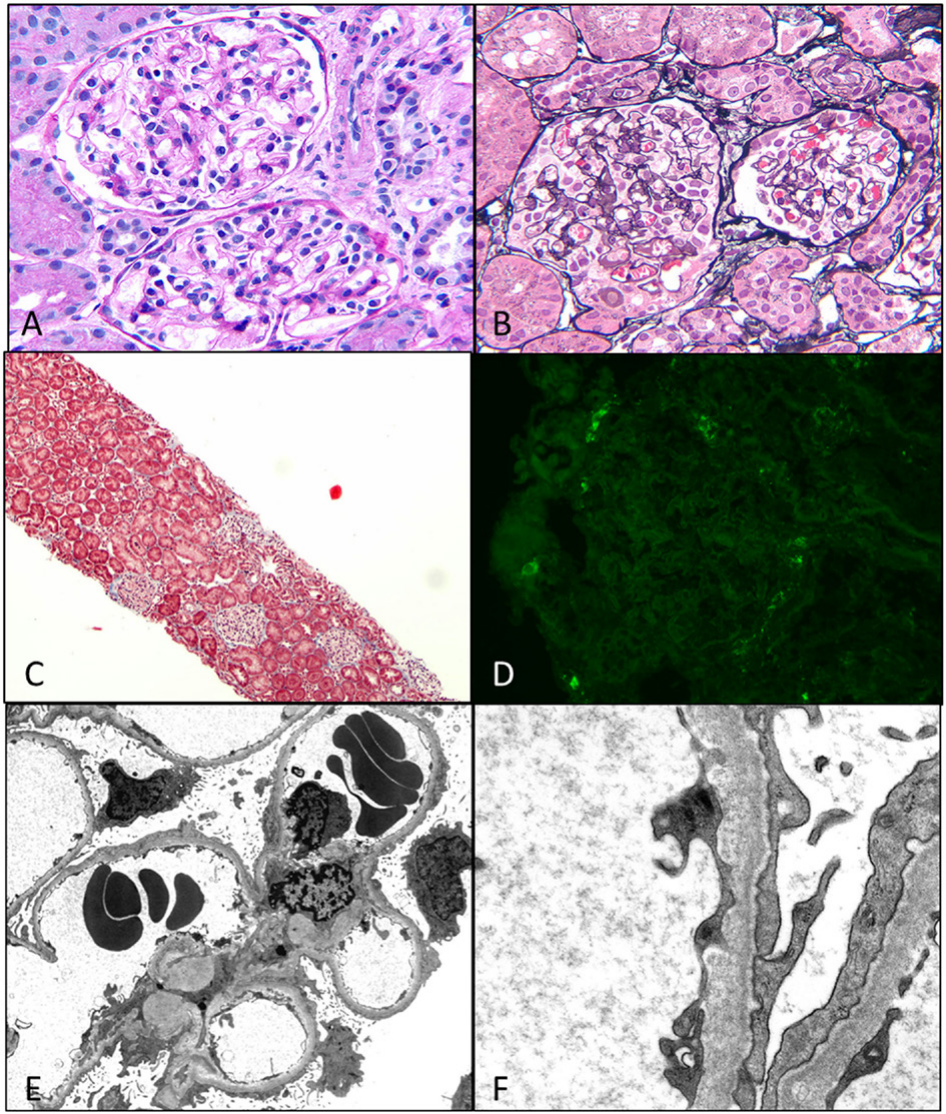
**Minimal change disease**. **(A)** A photomicrograph showing normal appearing uniform sized glomeruli in the cortex (H&E 400×). **(B)** A silver stain showing the normal loops with accentuation of the epithelial cells (Jones 400×). **(C)** A low magnification image of a trichrome stain showing no glomerular or interstitial fibrosis (Trichrome 40×). **(D)** A negative immunofluorescence panel for immunoglobulins or C3 (Direct immunofluorescence 400×). **(E,F)** An electron micrograph image showing open capillary loops with effacement of foot processes better visualized in **(F)**, which shows the diffuse fusion of foot processes [6200× **(E)** and 46,000× **(F)**].

Light microscopy usually shows normal glomeruli and tubules with the only change being prominence and swelling of visceral epithelial cells (2, 16). Patchy mild expansion of the mesangium may be seen in some cases (Figure 4). Histological finding of even a single glomerulus with segmental sclerosis, hyalinosis, or synechiae is enough to warrant a diagnosis of FSGS. Immunofluorescence is usually negative for any immune deposits, although there may be weak staining for IgM or C3 in a mesangial location in some cases. Albumin is positive within tubules reflecting the albuminuria. EM shows diffuse foot process effacement with prominent villous transformation, directly correlating with the severity of proteinuria and less prominent and patchy following treatment. Although this is the usual pattern, variants with increased mesangial hypercellularity are described. Immature glomeruli may be seen with this mesangial hypercellularity variant. In those patients presenting with acute renal failure, acute tubular injury and interstitial inflammation may be seen. Tubular atrophy is, however, not a feature of MCD. In those cases where IF shows deposits, the most likely ones are IgM (IgM nephropathy) or C1q (C1q nephropathy). In those where IgA is the dominant immunoglobulin, coexistent IgA nephropathy must be considered (19). The differential diagnosis for MCD is FSGS and as mentioned earlier, presence of any of the glomerular changes or foci of tubular atrophy in the absence of typical FSGS lesion, should raise the possibility of FSGS. Foot process effacement is not specific to MCD and can be seen in any cause of severe proteinuria. The debate over C1q being a specific subtype is still open as more recent data seems to suggest that though the initial response to therapy is poorer in this group, the overall long-term outcome is not different from cases of MCD.

The diffuse mesangial hypercellularity variant of MCD defined by presence of more than four mesangial cells per mesangial region affecting at least 80% of the glomeruli is a rarer cause of NS in children (16, 20). These patients can present with hematuria and hypertension unlike classic MCD children. IF shows mainly IgM and C3 in the mesangium, whereas EM shows paramesangial deposits besides the diffuse foot process effacement. This variant is usually associated with increased initial resistance to steroid therapy, but the overall remission rates are similar to MCD.

IgM nephropathy is another unusual variant of MCD characterized by at least 2+ staining intensity for IgM on IF (20). The distribution is mainly mesangial but could be membranous, but EM shows only a few small paramesangial deposits, suggesting that this deposition may be more of protein trapping rather than an immune complex disease. Initial steroid resistance is followed by remission (21).

### Focal Segmental Glomerulosclerosis

Focal segmental glomerulosclerosis is defined by the presence of segmental sclerotic lesions within glomeruli causing NS. It is the cause of NS in about 10–20% of cases in children. There is an association with low birth weight infants, especially for secondary FSGS (22). The causes are manifold and include primary idiopathic FSGS, genetic, and familial cases included under secondary FSGS, and secondary FSGS due to systemic diseases, which are more common in adults but can be seen in older children too (Table 3).

**Table 3:** **Common forms of familial FSGS**.

Gene (protein effected)	Inheritance	Typical age of onset	Distinguishing clinical features
NPHS1 (nephrin)	AR	Infancy	Congenital nephrotic syndrome (Finnish type); severe nephrosis leading to ESRD
NPHS2 (podocin)	AR	3 months to 5 years	10–20% of SRNS in children
WT1 (Wilms tumor 1)	AD	Child	Diffuse mesangial sclerosis/FSGS ± Wilms tumor or urogenital lesions
PLCε1 (phospholipase Cε1)	AR	4 months to 12 years	Diffuse mesangial sclerosis/FSGS
CD2AP (CD2-associated protein)	AR	<6 years	Rre, progresses to ESRD
INF2 (inverted formin 2)	AD	Teen/young adult	Mild nephrotic syndrome, but progressive CKD
ACTN4 (α-actinin 4)	AD	Any age	Mild nephrotic syndrome may develop progressive CKD
TRPC6	AD	Adult (age 20–35 years)	Nephrotic, progressive CKD
tRNA^Leu(UUR)^ gene	Mitochondrial DNA	Adult	May be associated deafness, diabetes, muscle problems, retinopathy (maternal inheritance)

### Primary (Idiopathic) FSGS

**Figure 5:**
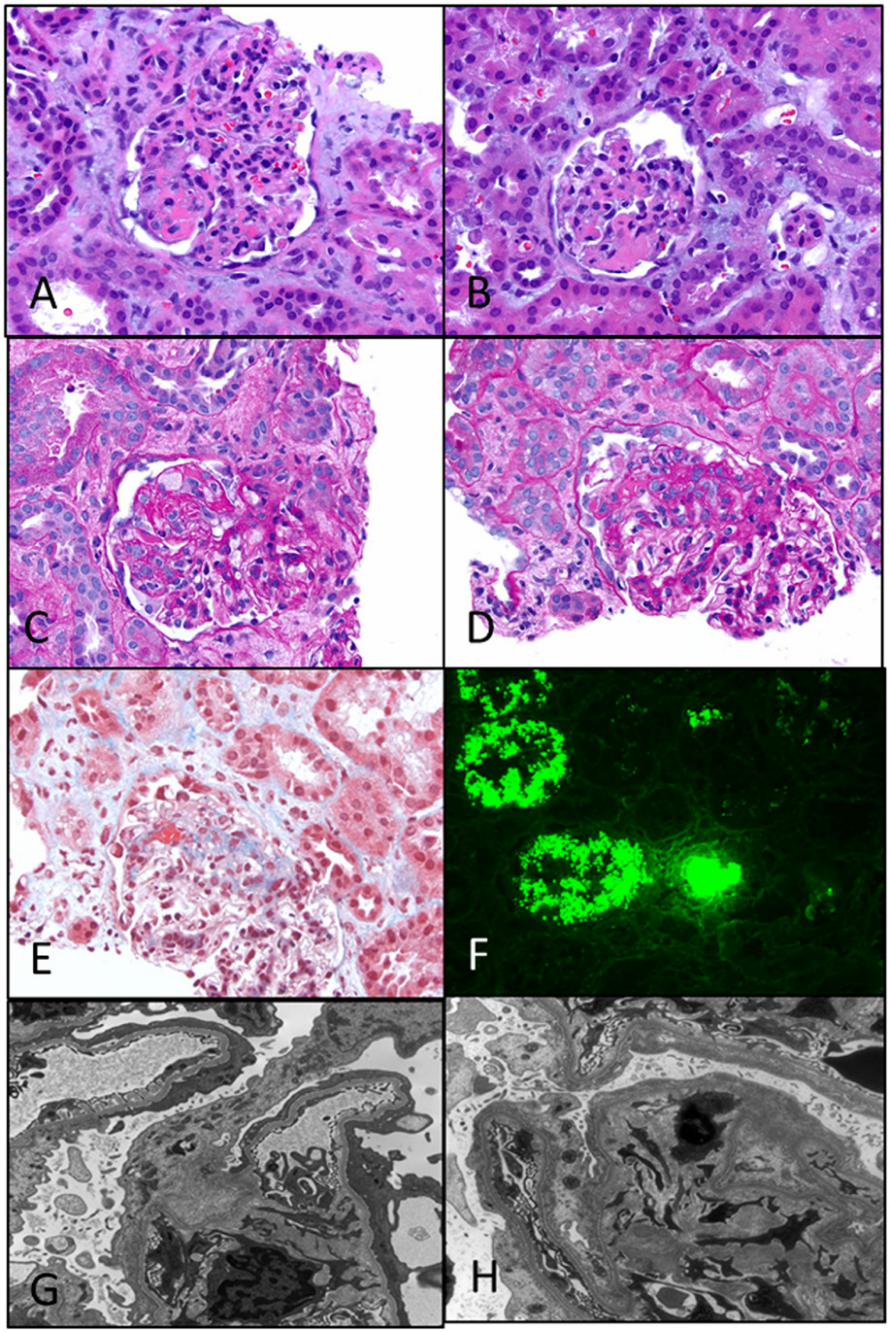
**Primary focal segmental glomerulosclerosis**. **(A,B)** Photomicrographs showing two glomeruli with a segmental lesion characterized by obliteration of capillary loops with solidification and eosinophilia of the segment due to sclerosis (H&E 200×). **(C,D)** PAS stained sections showing a focal sclerosing lesion in a segment in **(C)** and in the perihilar region in **(D)** close to the vascular pole. Note again the preservation of capillary loops in other segments of the glomeruli (PAS 200×). **(E)** A trichrome stain showing the perihilar zone of sclerosis as evidenced by the blue staining of that segment. Note also some fibrin deposition in that area (red) (Trichrome 200×). **(F)** Immunofluorescence showing strong staining for albumin in the tubules, a characteristic feature of nephrotic syndrome in general. Not shown is the associated IgG deposition (DIF 100×). **(G,H)** Two electron photomicrographs showing collapsed loops in the **(H)** with effacement of foot processes in both images. Typically, the foot process effacement may be segmental and over the sclerosed segments (EM 3400×).

This is the most common pattern of FSGS in children with a slight male preponderance and increased incidence in the African-American population (23). It presents with NS in 90% of cases but may present with renal insufficiency, hypertension, and hematuria in a proportion of cases. The histological variants described for FSGS include the classic or FSGS NOS variant, cellular variant, tip lesion, FSGS with mesangial hypercellularity, perihilar FSGS, and collapsing FSGS. Light microscopy for FSGS NOS is characterized by discrete segmental solidification of the glomerular tuft with a predilection for the juxtaglomerular region in the early stages of the disease. They commonly affect the vascular pole or the periphery of the tuft. The capillaries are occluded by acellular matrix material that produces intramembranous hyalinosis lesions highlighted as bright pink with a PAS stain and red with a trichrome stain, with progressive wrinkling of the membranes, development of adhesions and synechiae with the Bowman’s capsule with a prominence of visceral epithelial cells over this tuft (Figure 5). While focal lesions are the rule in early cases, with progression, glomerular obsolescence results. Tubular atrophy and interstitial fibrosis are evident in the vicinity of the sclerosed glomerulus involving the same nephron unit. Tubules contain protein resorption droplets and lipid droplets (24–26). IF usually shows focal and segmental granular IgM and C3, both within the membranes of the sclerosed segment and mesangium. Albumin will be noted in tubules. EM shows wrinkling and retraction of the glomerular basement membrane with accumulation of hyaline. There is complete effacement of foot processes overlying the sclerotic region with podocyte hypertrophy and focal microvillus transformation. Effacement may be mild to severe in the adjacent non-sclerotic glomeruli but affects more than half the surface membrane.

#### Cellular FSGS

This variant is characterized on light microscopy by segmental hypercellularity resembling focal proliferative GN with areas of the tuft showing endocapillary proliferation with luminal obliteration of capillaries, increased mesangial cells, and prominent increase in inflammatory cells, including neutrophils, foam cells, and monocytes, with marked podocyte hyperplasia giving the appearance of crescents (pseudocrescents). IF shows some IgM and C3, whereas EM shows no significant deposits but prominent foot process effacement with intact basement membranes.

#### Tip Lesion

This is characterized by proliferation of swollen podocytes and endocapillary proliferation with progressive obsolescence located at the tip of the tuft at the origin of the proximal tubule. As the lesion progresses, it results in a segmental scar in that area. There is associated tubular atrophy and interstitial fibrosis develops over time and may not be a feature in the early classic tip lesions. IF again shows IgM and C3, whereas EM resembles the cellular variant.

#### Primary FSGS with Mesangial Hypercellularity

In this variant, the histology reveals classic segmental sclerosing lesions associated with podocyte hyperplasia but with the associated finding of mesangial hypercellularity in the non-sclerotic glomeruli. The IF shows a diffuse presence of IgM and C3 in the mesangium of non-sclerotic glomeruli and in the zone of sclerosis. The EM shows extensive foot process effacement with no electron dense deposits.

#### Familial FSGS

Familial FSGS probably accounts for about 20% of cases of FSGS and can manifest at any age (27). The incidence increases to two-thirds of cases discovered in the first year of life. Several newer mutations have now been described to be associated with FSGS pattern of injury including mutations in the *NPHS2* gene (podocin defect, chromosome 1q25-31), with an autosomal-recessive mode of inheritance, actinin 4 defect (*ACTN4*, autosomal dominant), TRPC6 (transient receptor potential cation channel, subfamily C, member 6), and CD2AP (7, 28, 29). Of these the podocin defect is the most common and can be detected by immunohistochemistry that shows loss of podocin staining. Other mutations and their known associations are shown in Table 3.

#### Secondary FSGS

**Figure 6:**
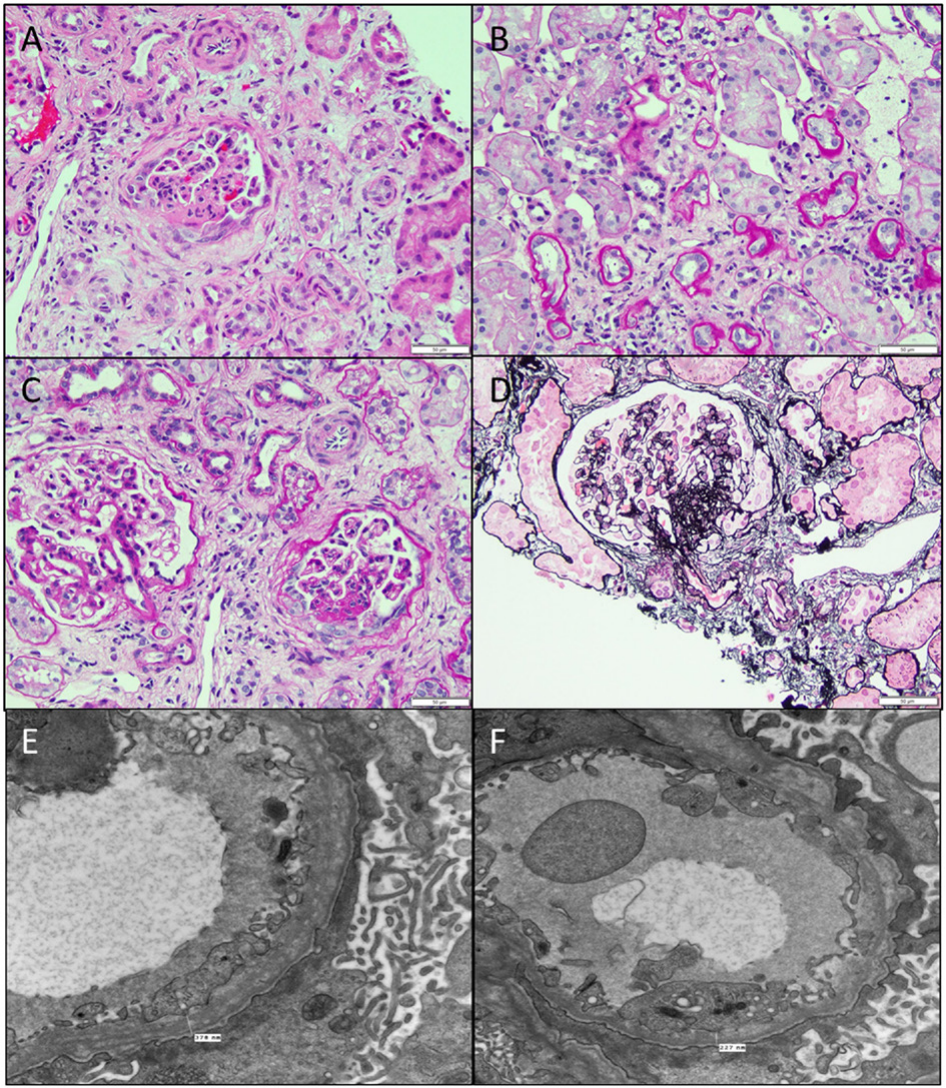
**Secondary FSGS**. **(A)** An image showing a biopsy with interstitial fibrosis, tubular atrophy, and a segmentally sclerosed glomerulus (H&E 100×). **(B)** PAS stain showing the extensive tubular atrophy (PAS 100×). **(C)** PAS stain showing two glomeruli, one larger than the other with the smaller one showing an area of sclerosis associated with epithelial cell proliferation in that area (PAS 200×). **(D)** A silver stain showing the segmental sclerosis in the perihilar region (Jones 200×). **(E,F)** Electron micrographs showing two images of the basement membranes with variable diameters showing prominent splitting of lamina densa with a basket weave appearance characteristic of hereditary nephritis, proven by collagen studies. Note also the microvillous transformation of foot processes and effacement in this patient with nephrotic range proteinuria (11,500×).

Segmental sclerosis can occur commonly in other diseases, not only in adults but also in children. The most common pediatric diseases that can have a component of associated NS in addition to a nephritic picture with histological evidence of FSGS include IgA nephropathy, Hereditary nephritis (Alport’s syndrome), and lupus nephritis The histological picture and immunofluorescence usually reveal the underlying disease, for example, mesangial hypercellularity and mesangial IgA in IgA nephropathy, full-house pattern in lupus nephritis, and thin basement membrane disease with basement membrane abnormalities on EM in Alport’s syndrome (Figure 6).

## Other Histological Variants of FSGS

### Collapsing Glomerulopathy

**Figure 7:**
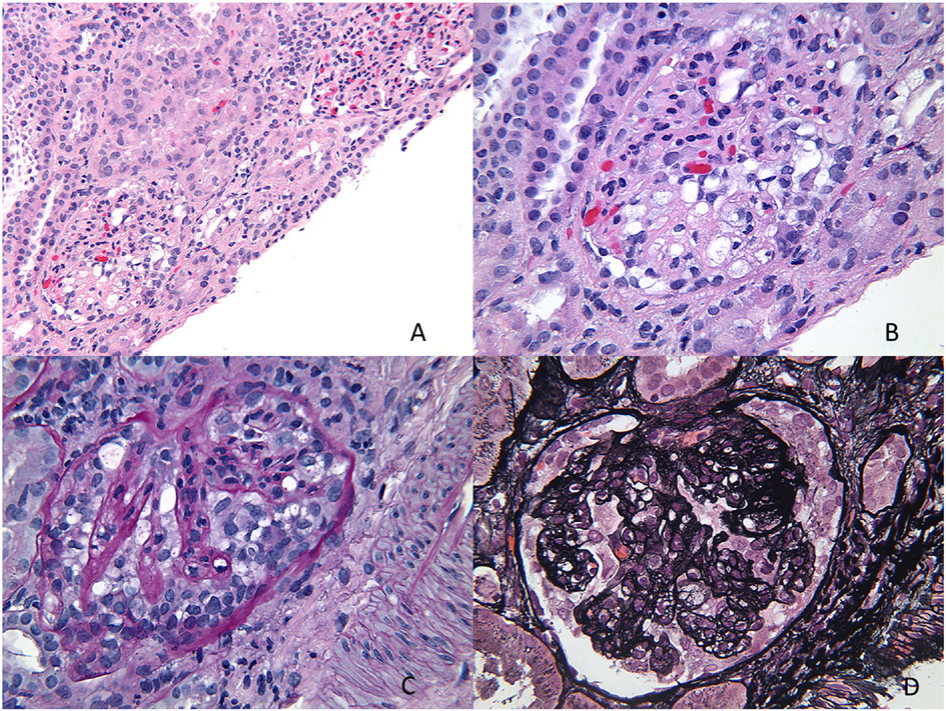
**Collapsing glomerulopathy**. **(A,B)** H&E images showing a low power and higher magnification appearance of a glomerulus with prominent epithelial cells and closed capillary loops better visualized on special stains (H&E 100× and 200×). **(C)** A PAS stain showing the collapsed cords of basement membranes with exuberant epithelial cell proliferation that seems to “choke” the capillary loops (PAS 400×). **(D)** A silver stain showing another glomerulus with wrinkling of the basement membranes and prominent epithelial cell proliferation (Jones 400×).

Collapsing variant of FSGS is characterized by progressive obliteration of capillary lumina due to wrinkling and shrinking of the basement membranes (23, 25, 30). It is relatively uncommon in children and was originally described in patients with HIV, but subsequently also seen in the absence of HIV. Other causes of collapsing glomerulopathy (CG) include infections, such as malaria and visceral leishmaniasis, drugs, such as interferon-alpha, bisphosphonates, and valproic acid, and autoimmune diseases, such as thrombotic microangiopathy and hematologic malignancies (31). They are as a group associated with progressive renal failure and have more severe disease at presentation. In children, rare forms associated with mitochondrial disorders have been reported. The COQ2 mutation is the most common and inherited as an autosomal-recessive condition, encoding for para-hydroxybenzoate-polyprenyl-transferase enzyme of the CoQ10 synthesis pathway (32). The MYH9 gene, an encoding non-muscle myosin IIA heavy chain gene, has been implicated in HIV associated CG and genetic FSGS in patients of African descent (33). A renal biopsy shows characteristic CG with glomerular epithelial cells showing abnormal mitochondria. It is important to recognize this variant, as it responds to ubiquinone replacement therapy. Histologically, CG is characterized by an implosive collapse of the capillary loops with wrinkling and contraction of the basement membrane with compensatory hypertrophy and hyperplasia of the podocytes, which tend to fill the Bowman’s space resembling crescents (Figure 7). There is no endothelial proliferation in this condition and no hyaline droplets or lipid is noted. Prominent tubulointerstitial changes are a feature. Typically, no significant deposits are noted in the membrane besides some IgM and C3. EM shows wrinkling with little to no thickening of the basement membranes with marked hypertrophy of the overlying podocytes. There is marked foot process effacement involving even the glomeruli without the collapsing lesions. In general, patients with CG progress rapidly to renal failure and show resistance to steroid therapy.

### C1q Nephropathy

**Figure 8:**
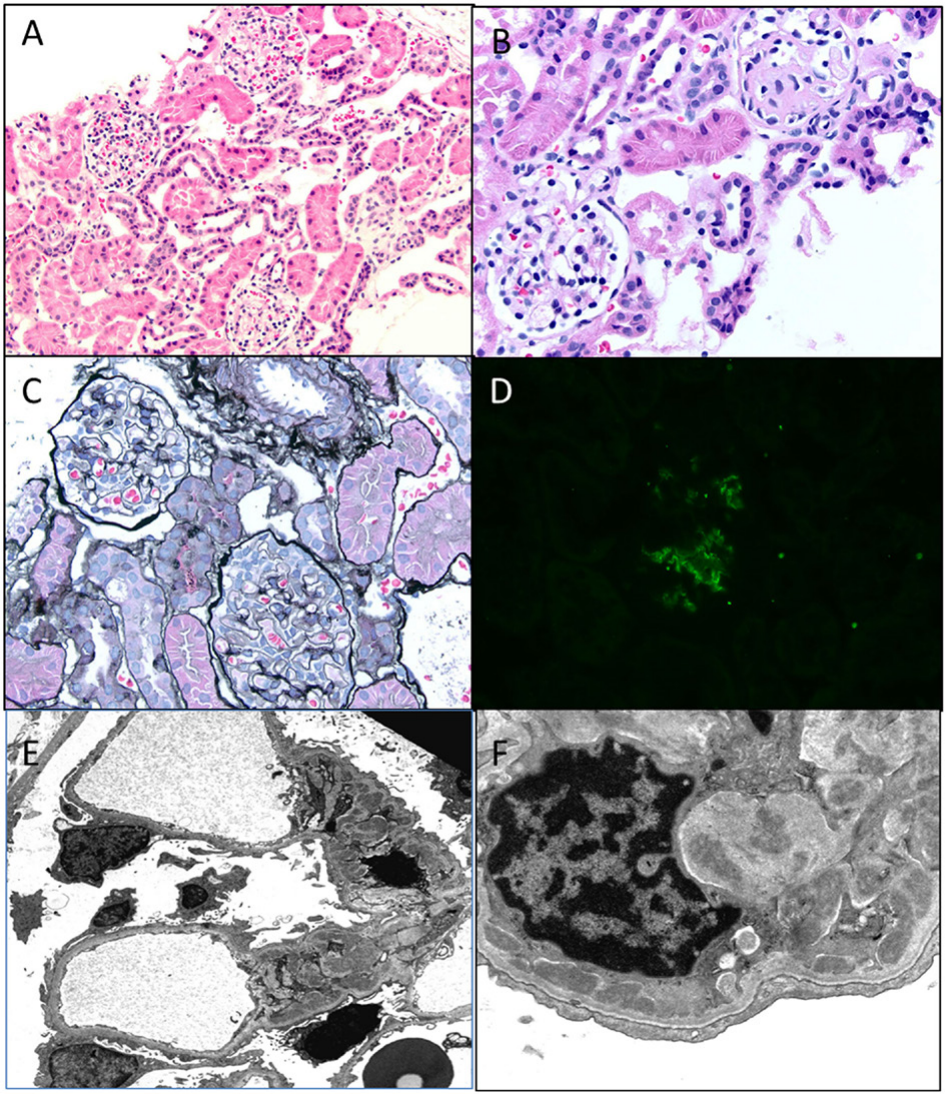
**C1q nephropathy**. **(A,B)** Light microscopy showing a low and high magnification images of normal appearing glomeruli in this child with nephrotic syndrome. Note one glomerulus in **(B)** is undergoing normal obsolescence (H&E 100× and 200×). **(C)** A silver stain showing normal glomeruli with no tubular atrophy (Jones 100×). **(D)** Immunofluorescence for C1q shows a 2+ staining in a mesangial location within a glomerulus. All other stains were negative, including C3, IgG, and IgM (DIF 100×). **(E,F)** Electron micrographs showing open capillary loops with mesangial, paramesangial, and even some subendothelial deposits. Note diffuse foot process effacement (3400× and 7100×).

As mentioned before, the existence of C1q nephropathy as a distinct subgroup of NS has been debated. The original description of this entity was by Jennette and Hipp (34). Although originally described as a form of FSGS, the histological spectrum for C1q nephropathy (Figure 8) could range from MCD to mild mesangial proliferation to FSGS (35). The clinical manifestations include nephrotic range proteinuria with or without hematuria, with hypertension and renal insufficiency in a subset of patients (36, 37). Serology is usually negative, and serum C3 and C4 levels are normal. Most patients seem to present with steroid-resistant NS. The hallmark is presence of strong (2+ and above) staining for C1q on IF in a mesangial pattern (35, 38). IgG and/or IgM may also be present to some extent as can IgA. EM would show features of either MCD or FSGS with some mesangial electron dense deposits. The deposits may be predominantly seen in a “paramesangial” location. Foot process fusion will also be noted (34, 35). The important differential diagnosis to exclude for this condition is lupus nephritis and IgA nephropathy. These two conditions are distinguished by their distinct immunofluorescence pattern and in the case of lupus nephritis with finding of tubuloreticular inclusions on EM. A membranoproliferative pattern of histology would also not favor a diagnosis of C1q nephropathy.

The exact significance of this entity is still debated with its relationship to outcome being variable. They do not necessarily portend a worse outcome as previously believed with a median renal survival in C1q nephropathy/FSGS being about 81 months (35). They may be, however, associated with relapses, steroid-resistant, and steroid-dependent NS (21).

In conclusion, the pathology of podocytopathies have some unique and some overlapping features and are frequently associated with specific genetic mutations. A summary of the pathological findings have been summarized in Table 4 for the reader.

**Table 4:** **Pathological features of podocytopathies in children**.

Diagnosis	Light microscopy glomeruli	LM tubulointerstitial changes	Immunofluorescence	Electron microscopy
CNS Finnish type	Normal immature glomeruli early; later mesangial increase; sclerosis over time	Early cystic changes in tubules, prominent over time. No casts, epithelium attenuated	Initial none; later IgM and C3	Foot process effacement. No deposits. Non-specific
DMS	Increased mesangial matrix diffuse; segmental sclerosis possible; podocyte hyperplasia	Tubular ectasia, small cysts, casts, progress to atrophy and interstitial fibrosis	IgM and C3 in mesangium	Extensive foot process effacement; increased mesangial matrix, no deposits. BM thickened and lamellated
Minimal change Disease (MCD)	Normal appearance of glomeruli, some podocyte prominence	Normal	Negative	Diffuse effacement of foot processes; partial if treated
FSGS cellular	Segmental hypercellularity – endothelial cells, foam cells, and inflammatory cells, podocyte hyperplasia – pseudocrescents	Atrophy variable with blood in tubules; interstitial inflammation	C3, IgM	Foot process effacement restricted. Capillary lumina occluded
FSGS NOS	Segmental sclerosis, podocyte hyperplasia, random distribution of sclerotic segment; hyalinosis	Atrophy with interstitial inflammation and fibrosis variable	C3 and IgM	Foot process effacement in region of sclerosis. No deposits usually but small paramesangial
FSGS with mesangial hypercellularity	Segmental sclerosis, mesangial hypercellularity of non-sclerotic glomeruli	Tubular atrophy and interstitial fibrosis	IgM and C3 in sclerosis and diffusely in mesangium	Extensive podocyte effacement segmental. No deposits
Collapsing glomerulopathy	Diffuse basement membrane wrinkling with collapse of capillary BM with obliteration of lumina, diffuse podocyte hyperplasia	Extensive, atrophy, interstitial inflammation, edema, fibrosis, tubular regeneration	IgM, C3	Wrinkling of BM, podocyte prominence, foot process effacement. Rare paramesangial deposits, tubuloreticular inclusions only in HIV
C1q nephropathy	Normal or FSGS	Normal or tubular atrophy. Variable interstitial changes	C1q at least 2+; IgG, IgM, C3	Mesangial and paramesangial deposits; rare subendothelial, foot process effacement

## Author Contributions

The author has reviewed the literature and provided a succinct account of the genetics and pathology of nephrotic syndrome in children.

## Conflict of Interest Statement

The author declares that the research was conducted in the absence of any commercial or financial relationships that could be construed as a potential conflict of interest.
